# Association between rheumatoid arthritis and chronic respiratory diseases in a Japanese population: A Mendelian randomization study

**DOI:** 10.1097/MD.0000000000039319

**Published:** 2024-08-16

**Authors:** Shaoning Shen, Hanbing Zeng, Hao Wei, Lianguo Wu

**Affiliations:** a Department of Orthopedics, The Second Affiliated Hospital of Zhejiang Chinese Medical University, Gongshu District, Hangzhou, Zhejiang Province, China.

**Keywords:** asthma, chronic obstructive pulmonary disease, chronic respiratory diseases, Japanese population, Mendelian randomization, rheumatoid arthritis

## Abstract

Past observational studies have documented an association between rheumatoid arthritis (RA) and chronic respiratory diseases. Undertaking the approach of Mendelian randomization (MR) analysis, this research aims to delve deeper into the probability of a causal connection between RA and chronic respiratory diseases. Collated genome-wide association study data covering RA with 4199 cases against 208,254 controls, asthma comprising 8216 cases versus 201,592 controls, and chronic obstructive pulmonary disease (COPD) detailing 3315 cases in contrast to 201,592 controls were derived from the repository of the Japanese Biobank. A selection of 10 RA-related, 8 asthma-related, and 4 COPD-related single nucleotide polymorphisms notable for their statistical significance (*P* < 5 × 10^−8^) were identified as instrumental variables. The primary analytical technique was the inverse variance-weighted (IVW) method, alongside the MR-Egger protocol, weighted median, and weighted mode to reinforce the validity and solidity of the principal results. For scrutinizing possible implications of horizontal pleiotropy, we harnessed the MR-Egger intercept examination and the Mendelian Randomization Pleiotropy REsidual Sum and Outlier test. Employing the inverse variance-weighted technique, we established a positive correlation between genetic predispositions for RA and actual occurrences of asthma (odds ratios [OR] = 1.14; 95% confidence intervals [CI]: 1.04–1.24; *P* = .003). This correlation remained strong when testing the results utilizing various methods, including the MR-Egger method (OR = 1.32; 95% CI: 1.09–1.60; *P* = .023), the weighted median (OR = 1.16; 95% CI: 1.06–1.26; *P* < .001), and the weighted mode (OR = 1.21; 95% CI: 1.11–1.32; *P* = .002). Furthermore, our findings from the inverse variance-weighted method also demonstrated a positive association between genetically predicted RA and COPD (OR = 1.12; 95% CI: 1.02–1.29; *P* = .021). However, no such link was discerned in supplementary analyses. In a shifted perspective—the reverse MR analysis—no correlation was identified between genetically predicted instances of asthma (IVW, *P* = .717) or COPD (IVW, *P* = .177) and RA. The findings confirm a causal correlation between genetically predicted RA and an elevated risk of either asthma or COPD. In contrast, our results offer no support to the presumed causal relationship between genetic susceptibility to either asthma or COPD and the subsequent development of RA.

## 1. Introduction

Rheumatoid arthritis (RA) is a systemic autoimmune disease characterized by progressive joint damage.^[1]^ Epidemiological studies have identified significant variations in RA prevalence amongst different regions and populations. The Global Burden of Disease study reports a prevalence of about 0.40% in Europe and 0.38% in North America,^[2]^ in contrast to 0.28% in China,^[3]^ and 0.75% estimated for Japan in 2020.^[4]^

Chronic respiratory diseases (CRD) encompass a spectrum of pulmonary conditions affecting the airways and other structures of the lung, and stand as a principal contributor to global morbidity and mortality. Chief among these are chronic obstructive pulmonary disease (COPD) and asthma, which play substantial roles in exacerbating the worldwide impact of noncommunicable diseases.^[5]^ A multitude of observational clinical studies have unearthed associations between RA and CRD.^[6,7]^ A prospective cohort study demonstrated an association of both asthma (HR 1.53 [95% confidence intervals [CI] 1.14–2.05]) and COPD (HR 1.89 [95% CI 1.31–2.75]) with an augmented risk of developing RA, independent of smoking or other potential confounders.^[8]^ Contrarily, another study identified a significant correlation between RA and COPD (HR = 1.68, 95% CI: 1.36–2.07), but not with asthma (HR = 1.11, 95% CI: 0.59–2.09),^[9]^ whereas additional research posits an increased RA risk correlated with asthma.^[10,11]^ Nonetheless, observational studies are unable to confirm causation owing to limitations such as unaccounted confounding variables or the possibility of reverse causality. Randomized controlled trials, the gold standard for establishing causality, present ethical challenges when applied to RA and chronic respiratory diseases.^[12–14]^ To this end, a definitive causal link between RA and chronic respiratory diseases remains to be confirmed.

Mendelian randomization uses genetic variants (such as single nucleotide polymorphisms [SNPs]) as instrumental variables (IVs) to assess the causal effect of an exposure on an outcome. Due to the randomness in the transmission of genetic variants from parent to offspring, these variants can serve as unconfounded proxies for the effect of altering a modifiable trait, similar to treatment allocation in a randomized controlled trial. This makes Mendelian randomization a credible approach for making causal inferences across various exposure-outcome pairs. With the rapid expansion of publicly available genome-wide association study (GWAS) summary statistics over the past decade, the method’s flexibility and applicability have increased significantly.^[15]^ To date, Mendelian randomization (MR) studies conducted in European populations have yielded various conclusions.^[16–19]^ Chen^[17]^ utilized summary statistics for asthma (56,167 cases and 352,255 controls) and RA (2843 cases and 5540 controls) from IEU open GWAS, employing 53 asthma-related SNPs and 40 RA-related SNPs as IVs. The findings indicated a causal relationship between genetic susceptibility to asthma and an increased risk of RA, yet found no evidence to support a causal relationship between RA genetic susceptibility and asthma. Conversely, another MR study provided evidence for a causal effect of RA genetic susceptibility on the risk for COPD and asthma.^[19]^ East Asian populations have a high incidence of RA, which endangers public health interests. Although well-conducted studies of MR in European populations have been conducted, these conclusions are divided. Besides, given the genomic variances and disease characteristics (RA, COPD, and asthma) between East Asian and European populations, these findings may not be directly transferable to East Asian populations. Critically, there is no relevant MR study based on East Asian populations, and the causal relationship between RA and CRD in East Asian populations is unclear.

The current study aims to utilize Mendelian randomization analysis within The Japanese Population to investigate the potential causal relationship between RA and CRD.

## 2. Patients and methods

### 2.1. Study design

**Figure 1.**
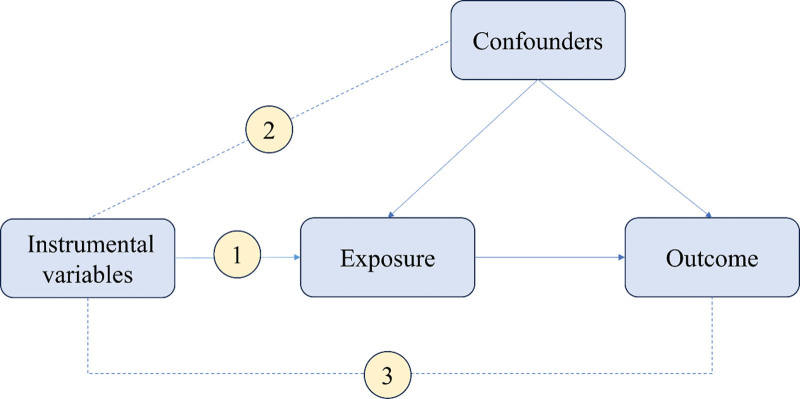
Assumptions and study design flowchart of the MR study. The MR method is based on 3 hypotheses: (1) instrumental variables directly affect the exposure; (2) instrumental variables are independent of any confounding factor; and (3) instrumental variables. MR = Mendelian randomization.

A bidirectional MR analysis was conducted to dissect the bidirectional causal effects between RA and chronic respiratory diseases, including asthma and COPD. An effective MR investigation requires adherence to 3 pivotal assumptions: (1) the IVs must satisfy the relevance criterion, being strongly linked to the exposure; (2) they should remain independent from confounding factors, upholding the independence criterion; (3) lastly, the exclusion-restriction criterion is crucial, positing that the IVs influence the outcome solely through the exposure. Figure 1 depicts this relationship between exposure and outcome.

### 2.2. Data source

The publicly available, summarized GWAS dataset on RA (4199 cases and 208,254 controls), COPD (3315 cases and 201,592 controls), and asthma (8216 cases and 201,592 controls) were sourced from the BioBank Japan (BBJ).^[20]^ Regarded as the largest East Asian biobank, the BBJ encompasses over 200,000 Japanese individuals aged 20 to 89 years, who were enlisted and monitored across 12 medical facilities from 2003 to 2018. The corresponding research involving BBJ was sanctioned by the Ethics Committee of the RIKEN Yokohama Institute and the Institute of Medical Science at the University of Tokyo, ensuring all study participants provided written informed consent.^[20]^ Diagnoses of RA, asthma, and COPD were conducted by expert physicians across the partner institutions. Subsequently, the BBJ compiled diagnostic information from medical records.

### 2.3. IVs selection

For our selection of SNPs to serve as IVs, we established stringent criteria: (1) a demonstrated strong association with exposure (*P* < 5 × 10^−8^), (2) confirmed independence from other genetic variables (linkage disequilibrium r^2^ < 0.001 within a 10,000 kb radius), (3)adequate effect allele frequencies (≥5%), (4) and an exclusion of palindromic SNPs with effect allele frequencies within the 40% to 70% range.^[12–14,21]^

### 2.4. Statistical analysis

Statistical analyses were performed using R version 4.3.2, utilizing both the TwoSampleMR package (version 0.5.6), and the MR-PRESSO package (version 4.2.0). The go-to technique for causal inference was the Inverse-variance weighted (IVW) analysis. In scenarios exhibiting SNP heterogeneity, a fixed-effect IVW model was deployed when Cochran Q-test *P*-value fell below the .05 threshold. Conversely, the random-effects IVW model was employed in its absence.^[22]^ To bolster the reliability of our outcomes, complementary methods such as MR-Egger, weighted median, and weighted mode were also integrated.^[23]^ Within MR analyses, a *P*-value <.05 signified indicative evidence for a potential link. The odds ratio (OR) and standard error were ascertained to quantify effect magnitudes. Furthermore, both IVW and MR-Egger regression analyses were leveraged to inspect result heterogeneity, measured using Cochran Q-test. The MR-Egger regression intercept functioned as a measure for detecting prospective horizontal pleiotropy.^[24]^ The MR-PRESSO test was designated to identify aberrant SNPs associated with horizontal pleiotropy. The “leave-one-out” analysis was utilized, excising individual SNPs that may independently sway MR estimations substantially.^[24]^ A query was conducted in the Phenoscanner database to confirm that the included SNPs were unrelated to known confounders. The potency of the IVs was evaluated via the F-statistic, with a strong instrument validity threshold delineated by an F-statistic exceeding 10.^[25]^ Furthermore, considering the exclusive derivation of GWAS data from the BBJ, apprehensions concerning winner’s curse and weak instrument bias attributable to sample overlap were acknowledged. Consequently, a stringent GWAS significance criterion *P* < 1 × 10^‐13^ was instituted, supplemented by additional sensitivity analyses. This threshold was instituted to mitigate the potential effects of the winner’s curse and the propensity of weak instrument bias, compared to the conventional benchmarks.^[21,26]^

## 3. Results

### 3.1. Strength of genetic instruments

In this investigation, we initially considered a total of 12 SNPs associated with RA, 11 SNPs linked to asthma, and 6 SNPs connected to COPD. One SNP with palindromic intermediate allele frequency related to RA, 2 SNPs with palindromic intermediate allele frequency related to asthma, 1 SNP related to asthma that is susceptible to confounding factors, and 2 SNPs with palindromic intermediate allele frequency related to COPD were excluded. Additionally, 1 SNP (rs117530403) related to RA with a significant independent effect (MR-PRESSO test, *P* < .011) was excluded from the conventional analysis, but it was retained in the strict threshold analysis as its impact was no longer pronounced. Finally, 10, 8, and 4 SNPs were consequently selected for the MR analyses targeting RA, asthma, and COPD, respectively. Comprehensive details are tabulated in Table 1.

**Table 1:** Characteristics of SNPs significantly associated with RA/asthma/COPD in The Japanese Population.

Exposure	SNP	CHR	Position	EA/NEA	β	EAF	SE	*P* value	F
RA									70.64
	rs12612769	2	191,953,998	C/A	0.150	0.303	0.025	1.06e‐09	37.2
	rs1557549	6	32,452,282	G/A	‐0.494	0.129	0.040	5.64e‐35	152.23
	rs1634734	6	31,307,885	A/G	‐0.176	0.450	0.023	6.27e‐15	60.81
	rs2082260	6	32,946,854	T/G	0.128	0.541	0.022	7.48e‐09	33.41
	rs3757387	7	128,576,086	C/T	0.212	0.107	0.036	4.83e‐09	34.26
	rs56139217	10	64,063,077	C/T	0.212	0.114	0.037	1.26e‐08	32.39
	rs77117142	6	25,462,110	T/C	‐0.343	0.057	0.049	3.97e‐12	48.14
	rs79658451	6	34,163,084	C/G	0.207	0.116	0.035	4.19e‐09	34.53
	rs80202727	6	44,249,164	T/C	0.186	0.239	0.027	2.47e‐12	49.07
	rs9275610	6	32,683,750	C/T	‐0.360	0.328	0.024	2.69e‐49	217.83
	rs117530403	6	32,566,482	T/G	1.028	0.134	0.039	4.76e‐153	694.47*
Asthma									45.16
	rs10519067	15	61,068,347	A/G	‐0.134	0.210	0.020	1.68e‐11	45.32
	rs10797119	9	92,202,495	C/T	0.102	0.323	0.018	1.45e‐08	32.11
	rs11256018	10	9,044,610	T/C	‐0.133	0.505	0.016	1.74e‐16	67.88
	rs1837253	5	110,401,872	C/T	0.128	0.352	0.017	3.67e‐14	57.34
	rs3024577	16	27,358,203	G/A	0.144	0.714	0.018	7.67e‐16	64.95
	rs3857440	5	131,794,069	A/G	0.098	0.315	0.017	2.07e‐08	31.42
	rs4449174	2	242,702,538	G/C	‐0.151	0.157	0.027	1.45e‐08	32.12
	rs75535961	2	204,603,769	A/G	‐0.088	0.500	0.016	4.06e‐08	30.12
COPD									43.67
	rs1529672	3	25,520,582	A/C	‐0.153	0.342	0.027	7.74e‐09	33.34
	rs1585258	4	89,879,196	T/G	0.159	0.651	0.027	3.13e‐09	35.1
	rs56129017	19	41,416,948	T/C	0.257	0.266	0.030	3.00e‐17	71.35
	rs59459325	19	41,175,390	T/C	‐0.202	0.168	0.034	3.52e‐09	34.87

COPD = chronic obstructive pulmonary disease, RA = rheumatoid arthritis, SNP = single nucleotide polymorphisms.

* This SNP was only included in the GWAS analysis with a stricter threshold.

### 3.2. Effects of RA on asthma

**Figure 2.**
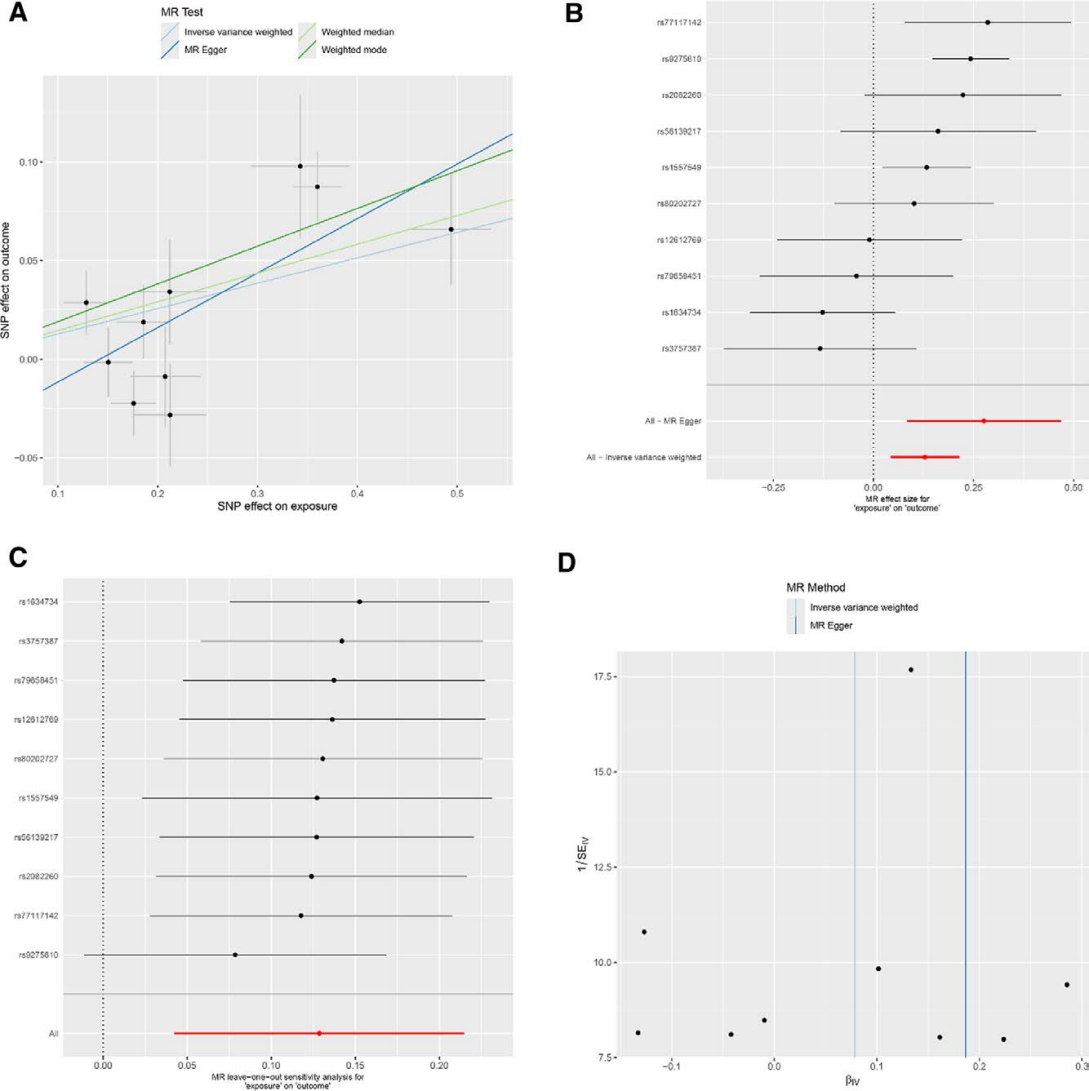
(Using 10 SNPs as IVs) (A) Scatter plot for casual effects of RA on asthma. The slope of each line represents an estimate of the effect of a different method using MR. (B) The forest plot analysis assessed the causal association between RA and asthma. (C) The leave-one-out sensitivity analysis assessed the causal association between RA and asthma. (D) Funnel plot of causality between RA and asthma. IVs = instrumental variables, MR = Mendelian randomization, RA = rheumatoid arthritis, SNP = single nucleotide polymorphisms.

**Figure 3.**
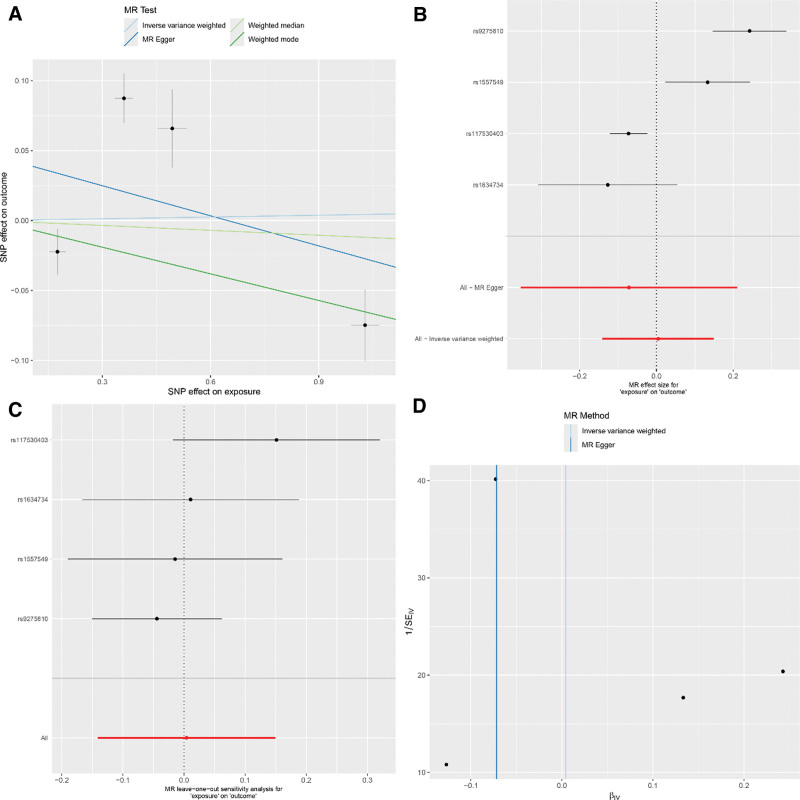
(Using 4 SNPs as IVs) (A) Scatter plot for casual effects of RA on asthma. The slope of each line represents an estimate of the effect of a different method using MR. (B) The forest plot analysis assessed the causal association between RA and asthma. (C) The leave-one-out sensitivity analysis assessed the causal association between RA and asthma. (D) Funnel plot of causality between RA and asthma. IVs = instrumental variables, MR = Mendelian randomization, RA = rheumatoid arthritis, SNP = single nucleotide polymorphisms.

The IVW method presented a notable link between the genetic predisposition to RA and the enhanced risk of developing asthma (IVW: OR = 1.14; 95% CI: 1.04–1.24; *P* = .003). This assertion was corroborated by additional methods including MR-Egger, weighted median, and weighted mode (MR-Egger: OR = 1.32; 95% CI: 1.09–1.60; *P* = .023; weighted median: OR = 1.16; 95% CI: 1.06–1.26; *P* < .001; weighted mode: OR = 1.21; 95% CI: 1.11–1.32; *P* = .002). The MR-Egger approach did not suggest any appreciable horizontal pleiotropy (intercept = ‐0.039; *P* = .138), and Cochran Q-test alluded to heterogeneity in the RA-asthma risk association. For detailed sensitivity analysis results, refer to Figure 2. Intriguingly, after enforcing a more rigorous GWAS threshold *P* < 1 × 10^‐13^, the number of instrumental SNPs leveraged in the analysis was refined to 4. Post adjustment, all MR methods reported no substantial association between RA and asthma (IVW: OR = 1.00; 95% CI: 0.87–1.16; *P* = .954; MR-Egger: OR = 0.93; 95% CI: 0.70–1.23; *P* = .668; weighted median: OR = 0.99; 95% CI: 0.93–1.05; *P* = .721; weighted mode: OR = 0.94; 95% CI: 0.89–0.99; *P* = .093). For detailed sensitivity analysis results, refer to Figure 3. These findings are detailed in Table 2.

**Table 2:** Mendelian randomization estimates for the association between rheumatoid arthritis and asthma.

Exposure	Outcome	No. SNPs	Method	OR (95%CI)	*P*-val	Q	Q–*P*-val	Intercept	Pleiotropy-*P*-val
RA (5e‐8)	Asthma	10	IVW	1.14 (1.04–1.24)	.003	23.752	0.005		
			MR Egger	1.32 (1.09–1.60)	.023	17.757	0.023	‐0.039	0.139
			Weighted median	1.16 (1.06–1.26)	.001				
			Weighted mode	1.21 (1.11–1.32)	.002				
RA (1e‐13)	Asthma	4	IVW	1.00 (0.87–1.16)	.954	40.397	8.8e‐09		
			MR Egger	0.93 (0.70–1.23)	.668	33.466	5.4e‐08	0.046	0.585
			Weighted median	0.99 (0.93–1.05)	.721				
			Weighted mode	0.94 (0.89–0.99)	.093				

IVW = inverse variance-weighted, RA = rheumatoid arthritis, MR = Mendelian randomization, SNP = single nucleotide polymorphisms.

### 3.3. Effects of RA on COPD

**Figure 4.**
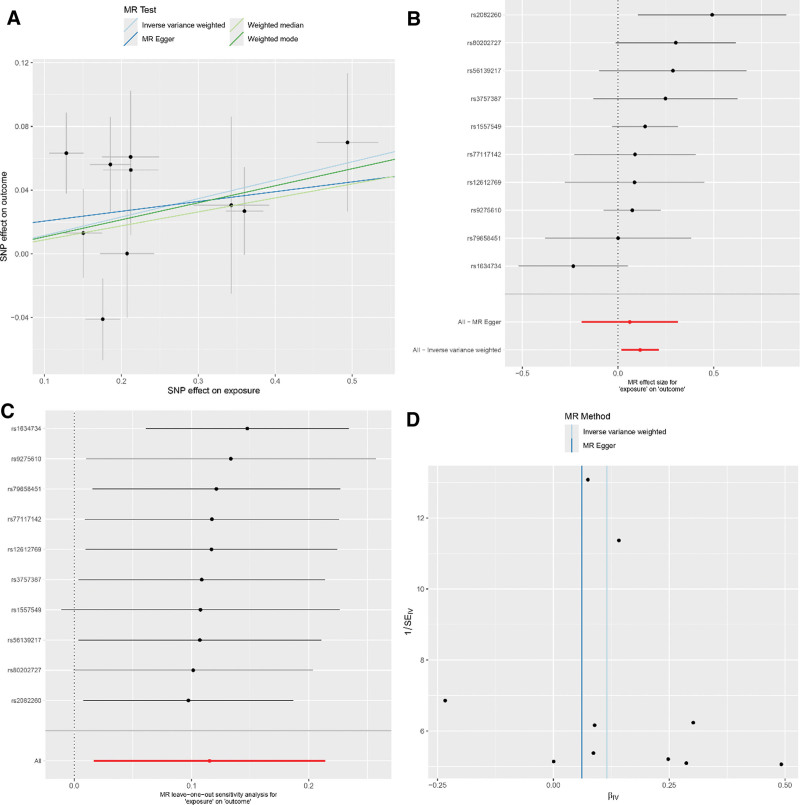
(Using 10 SNPs as IVs) (A) Scatter plot for casual effects of RA on COPD. The slope of each line represents an estimate of the effect of a different method using MR. (B) The forest plot analysis assessed the causal association between RA and COPD. (C) The leave-one-out sensitivity analysis assessed the causal association between RA and COPD. (D) Funnel plot of causality between RA and COPD. COPD = chronic obstructive pulmonary disease, IVs = instrumental variables, MR = Mendelian randomization, RA = rheumatoid arthritis, SNP = single nucleotide polymorphisms.

**Figure 5.**
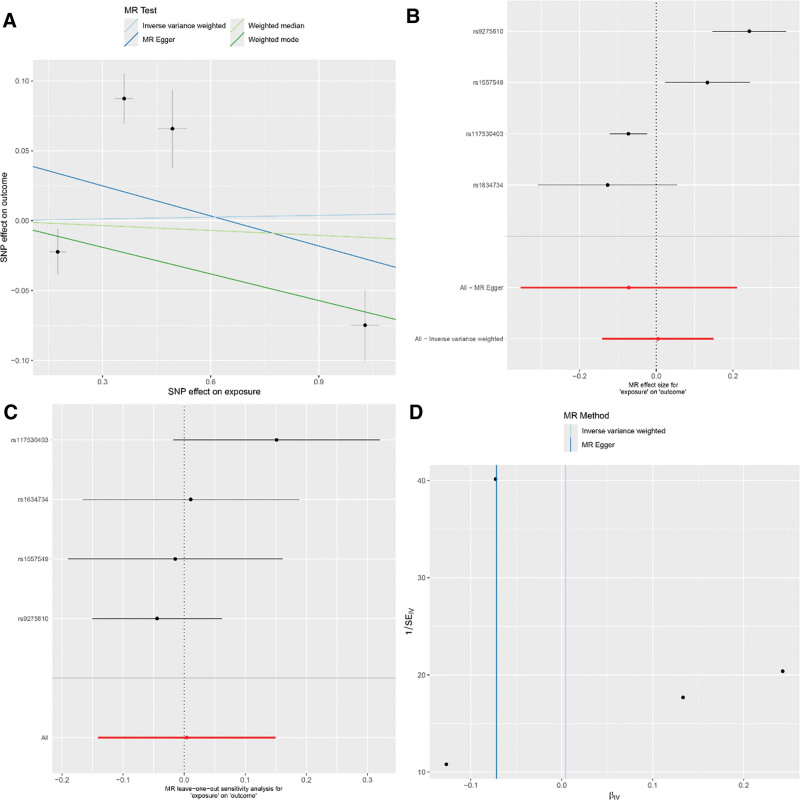
(Using 4 SNPs as IVs) (A) Scatter plot for casual effects of RA on COPD. The slope of each line represents an estimate of the effect of a different method using MR. (B) The forest plot analysis assessed the causal association between RA and COPD. (C) The leave-one-out sensitivity analysis assessed the causal association between RA and COPD. (D) Funnel plot of causality between RA and COPD. COPD = chronic obstructive pulmonary disease, IVs = instrumental variables, MR = Mendelian randomization, RA = rheumatoid arthritis, SNP = single nucleotide polymorphisms.

Our results demonstrate evident variability in the causal connections between RA and COPD when assayed via 5 different MR methods. The IVW method disclosed a meaningful link between genetic predilection to RA and an augmented risk for COPD (IVW: OR = 1.12; 95% CI: 1.02–1.24; *P* = .022). Nevertheless, subsequent tests did not establish a significant bond between RA and COPD (MR-Egger: OR = 1.06; 95% CI: 0.83–1.37; *P* = .644; weighted median: OR = 1.09; 95% CI: 0.98–1.22; *P* = .120; weighted mode: OR = 1.11; 95% CI: 0.99–1.25; *P* = .113). The MR-Egger procedure suggested an absence of horizontal pleiotropy (intercept = 0.014; *P* = .657), and Cochran Q test substantiated no heterogeneity in the assessed risk between RA and COPD. For detailed sensitivity analysis results, refer to Figure 4. Moreover, upon implementing a more stringent GWAS threshold *P* < 1 × 10^‐13^—which included the use of 4 SNPs as IVs—the entirety of the MR techniques concurred on the absence of an association between RA and COPD (IVW: OR = 0.97; 95% CI: 0.86–1.08; *P* = .539; MR-Egger: OR = 0.95; 95% CI: 0.75–1.20; *P* = .707; weighted median: OR = 0.97; 95% CI: 0.90–1.04; *P* = .339; weighted mode: OR = 0.94; 95% CI: 0.86–1.02; *P* = .224). For detailed sensitivity analysis results, refer to Figure 5. These specifics are depicted in Table 3.

**Table 3:** Mendelian randomization estimates for the association between RA and COPD.

Exposure	Outcome	No. SNPs	Method	OR (95%CI)	*P*-val	Q	Q–*P*-val	Intercept	Pleiotropy-*P*-val
RA (5e‐8)	COPD	10	IVW	1.12 (1.02–1.24)	.022	12.729	0.175		
			MR Egger	1.06 (0.83–1.37)	.644	12.399	0.134	0.014	0.657
			Weighted median	1.09 (0.98–1.22)	.120				
			Weighted mode	1.11 (0.99–1.25)	.113				
RA (1e‐13)	COPD	4	IVW	0.97 (0.86–1.08)	.539	9.556	0.022		
			MR Egger	0.95 (0.75–1.20)	.707	9.410	0.009	0.011	0.876
			Weighted median	0.97 (0.90–1.04)	.339				
			Weighted mode	0.94 (0.86–1.02)	.224				

COPD = chronic obstructive pulmonary disease, IVW = inverse variance-weighted, RA = rheumatoid arthritis, MR = Mendelian randomization, SNP = single nucleotide polymorphisms.

### 3.4. Effect of asthma or COPD on RA

**Figure 6.**
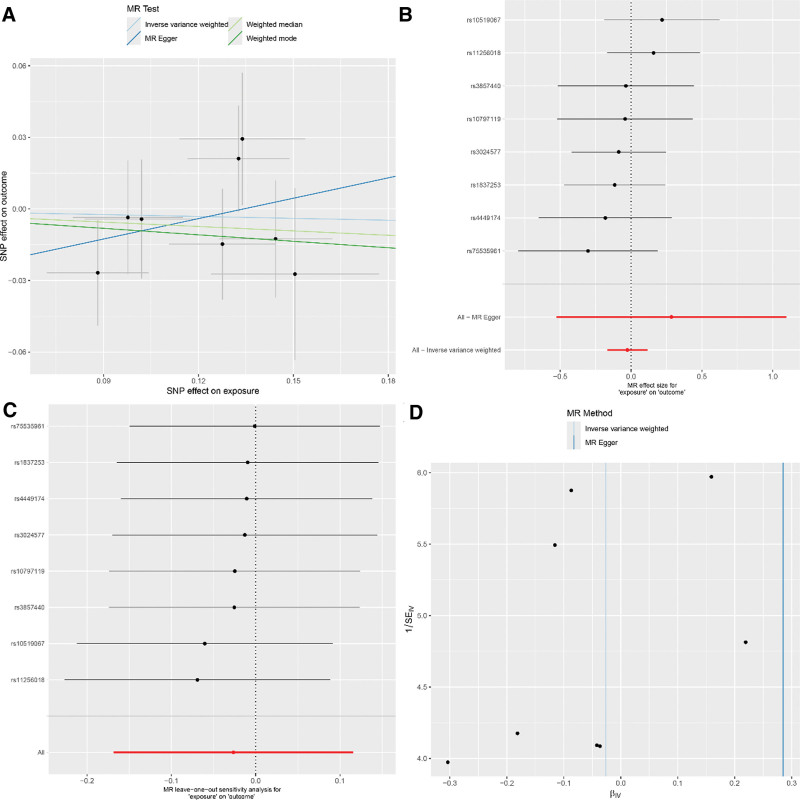
(A) Scatter plot for casual effects of asthma on RA. The slope of each line represents an estimate of the effect of a different method using MR. (B) The forest plot analysis assessed the causal association between asthma and RA. (C) The leave-one-out sensitivity analysis assessed the causal association between asthma and RA. (D) Funnel plot of causality between asthma and RA. MR = Mendelian randomization, RA = rheumatoid arthritis.

The reverse MR analysis evaluating the influence of asthma on RA risk incorporated 8 SNPs. The IVW method revealed an absence of association between the genetic inclination toward asthma and RA risk (IVW: OR = 0.97; 95% CI: 0.85–1.12; *P* = .717). Complementary methods, namely MR-Egger, weighted median, and weighted mode, were in agreement with this result (MR-Egger: OR = 1.33; 95% CI: 0.59–3.00; *P* = .517; weighted median: OR = 0.94; 95% CI: 0.79–1.13; *P* = .504; weighted mode: OR = 0.91; 95% CI: 0.69–1.21; *P* = .545). Analysis via MR-Egger detected no significant horizontal pleiotropy (intercept = ‐0.038; *P* = .474), and Cochran Q test confirmed no heterogeneity regarding asthma and RA risk. For detailed sensitivity analysis results, refer to Figure 6.

**Figure 7.**
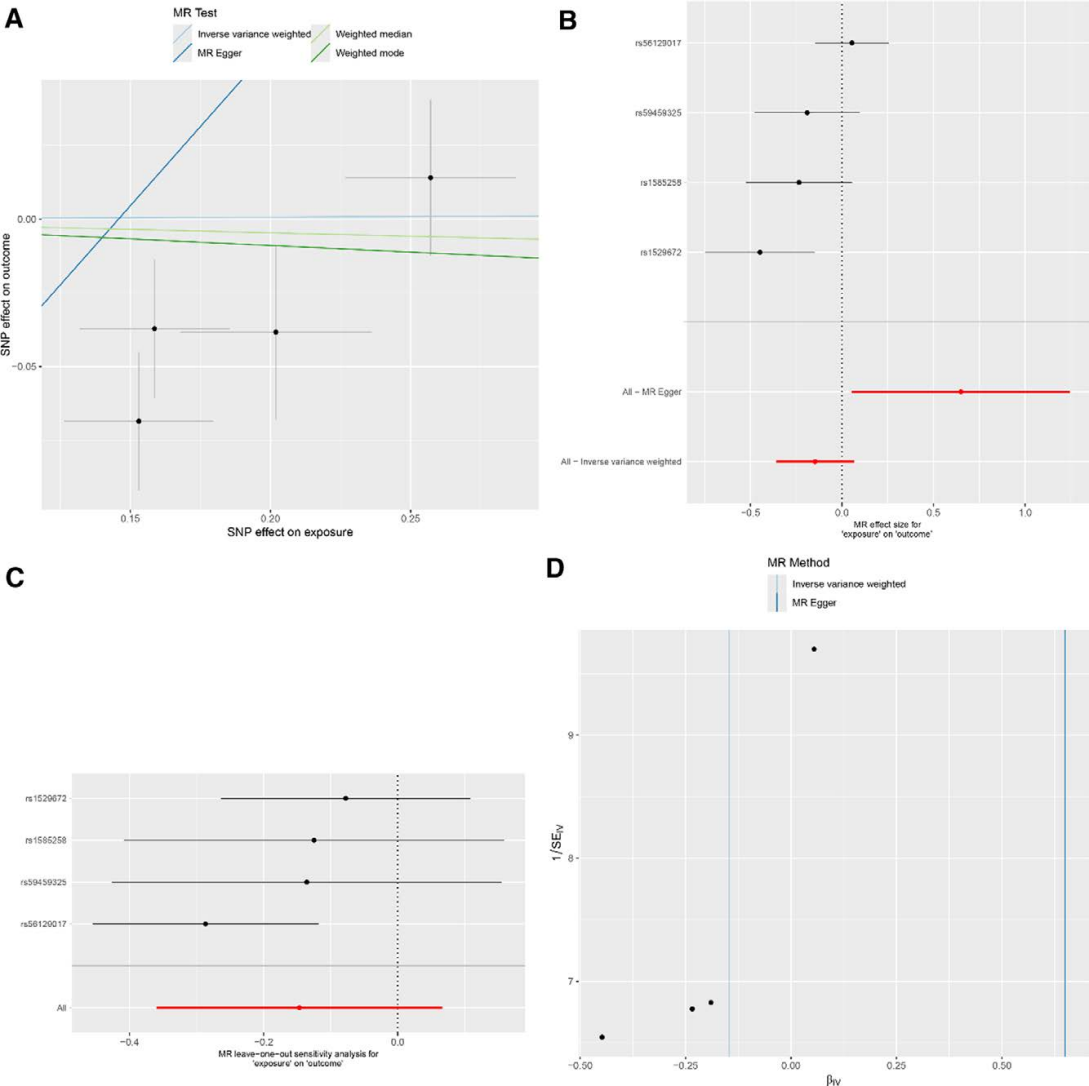
(A) Scatter plot for casual effects of COPD on RA. SNP: single nucleotide polymorphism. The slope of each line represents an estimate of the effect of a different method using MR. (B) The forest plot analysis assessed the causal association between COPD and RA. (C) The leave-one-out sensitivity analysis assessed the causal association between COPD and RA. (D) Funnel plot of causality between COPD and RA. COPD = chronic obstructive pulmonary disease, MR = Mendelian randomization, RA = rheumatoid arthritis.

**Figure 8.**
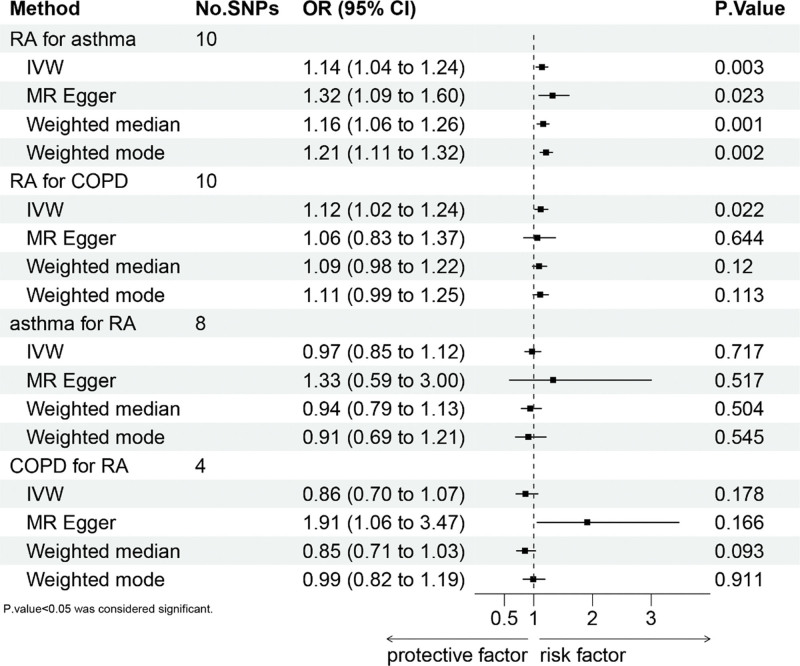
The association between RA and asthma or COPD. COPD = chronic obstructive pulmonary disease, RA = rheumatoid arthritis.

In the reverse MR scrutiny of COPD’s effect on RA risk, 4 SNPs were incorporated. Mirroring findings related to asthma, the IVW method did not demonstrate a link between genetic predictions of COPD and RA risk (IVW: OR = 0.86; 95% CI: 0.70–1.07; *P* = .178). Aligning with this, other methodologies yielded congruent outcomes (MR-Egger: OR = 1.91; 95% CI: 1.06–3.47; *P* = .166; weighted median: OR = 0.85; 95% CI: 0.71–1.03; *P* = .093; weighted mode: OR = 0.99; 95% CI: 0.82–1.19; *P* = .911). The MR-Egger examination indicated no horizontal pleiotropy (intercept = ‐0.157; *P* = .115), and Cochran Q test revealed an absence of heterogeneity between COPD and RA risk. For detailed sensitivity analysis results, refer to Figure 7. These particulars are delineated in Table 4. Summarized data for all the analyses are depicted in Figure 8.

**Table 4:** Mendelian randomization estimates for the association between asthma or COPD and rheumatoid arthritis.

Exposure	Outcome	No. SNPs	Method	OR (95%CI)	*P*-val	Q	Q–*P*-val	Intercept	Pleiotropy-*P*-val
Asthma	RA	8	IVW	0.97 (0.85–1.12)	.717	4.628	0.705		
			MR Egger	1.33 (0.59–3.00)	.517	4.046	0.670	‐0.038	0.474
			Weighted median	0.94 (0.79–1.13)	.504				
			Weighted mode	0.91 (0.69–1.21)	.545				
COPD	RA	4	IVW	0.86 (0.70–1.07)	.178	8.140	0.043		
			MR Egger	1.91 (1.06–3.47)	.166	0.936	0.626	‐0.157	0.115
			Weighted median	0.85 (0.71–1.03)	.093				
			Weighted mode	0.99 (0.82–1.19)	.911				

COPD = chronic obstructive pulmonary disease, IVs = instrumental variables, RA = rheumatoid arthritis, MR = Mendelian randomization, SNP = single nucleotide polymorphisms.

## 4. Discussion

This MR study was conducted to explore the potential causal connection between RA and chronic respiratory diseases in The Japanese Population. The data suggest a positive correlation between the genetic likelihood of RA and an elevated risk of asthma within the Japanese demographic. These conclusions are coherent across alternative MR techniques such as MR-Egger, weighted median, and weighted mode. A similar positive linkage was discerned between genetic predispositions to RA and an increased probability of developing COPD in the same population. Nonetheless, this relationship did not reach statistical significance when assessed via other MR method (MR-Egger, weighted median, and weighted mode). Conversely, in reverse MR analyses, no causative association was identified between asthma or COPD and the susceptibility to RA.

Notably, when using a more stringent threshold of *P* < 1 × 10^‐13^, there was no association observed between RA and asthma or COPD. In the conventional analysis, rs117530403 was excluded from the IVs due to its significant independent effect (MR-PRESSO test, *P* < .011). However, with the threshold set at *P* < 1 × 10^‐13^, this independent effect was no longer significant, so it was included as an IV. We believe that the primary reason for the difference in results is the heterogeneity in the GWAS analysis with *P* < 1 × 10^‐13^. The Forest plot and leave-one-out plot showed the significant negative impact of rs117530403 on the results. Based on this, we conservatively adopt the conventional analysis results as the primary findings of this study.

To our knowledge, there have been 4 MR studies based on European populations assessing the potential causal relationship between RA and asthma or COPD^[16–19]^ Yu et al’s MR study,^[16]^ based on the FinnGen database (COPD) and IEU database (RA), indicated a significant association between RA and the risk of developing COPD (OR, 377.313, 95% CI, 6.625–21,487.932, *P* = .004). The datasets from Cao et al’s MR study^[19]^ were derived from the European cohort GWAS meta-analysis (RA) and the FinnGen database (asthma and COPD). Their results indicated that the genetic susceptibility to RA influences the increased risk of asthma/COPD (OR = 1.03; 95% CI: 1.02–1.04, *P* = .018). Chen et al’s MR analysis^[17]^ based on the IEU Open GWAS indicated no causal relationship between RA and asthma (OR = 1.01; 95% CI: 0.98–1.03, *P* = .673). Yan et al’s MR analysis^[18]^ data were sourced from the European Bioinformatics Institute Consortium (RA) and the UK Biobank (asthma). The study results indicate that there is no causal relationship between RA and the risk of asthma occurrence (OR = 0.994, 95% CI: 0.987–1.001, *P* = .121).Genome and disease characteristics differences may partly explain the differences between previous studies and our research. We have compared our SNPs with those in the 4 European MR studies, and only 1 SNP in 1 of the studies overlapped with ours, none of the SNPs from the remaining studies overlapped with our research. This suggests that effective genetic variation may differ among different races. Moreover, considering that the underlying mechanisms may differ between mild to moderate asthma and severe asthma,^[27]^ but most GWAS data did not specifically categorize asthma subtypes, this has also led to differences in MR analysis results.

Earlier epidemiological inquiries have identified a comparatively higher incidence of asthma within individuals diagnosed with RA as opposed to the general population. Kim et al^[10]^ utilized data sourced from the Health Insurance Review and Assessment Service—National Sample Cohort (HIRA-NSC) in Korea, spanning the period of 2002 to 2013. This dataset comprised adult participants aged over 20, with a sample of 6695 RA patients and 26,780 control subjects, to appraise the historical occurrence of asthma. Results from this cohort highlighted a more prominent hazard ratio for asthma presence in the RA group in contrast to the control group (HR = 1.23, 95% CI = 1.15–1.32). A parallel cohort study conducted in Taiwan deduced a similar pattern,^[11]^ documenting that the incidence rate of asthma within the RA cohort was more than double the rate observed in the non-RA cohort (HR = 2.07). In 2013, Nannini et al were pioneers in reporting that individuals afflicted with RA also showed a heightened prevalence of COPD when measured against the general populace (HR: 1.54, 95% CI 1.01–2.34).^[28]^ Subsequent research efforts have reinforced this linkage,^[7,29–31]^ with a meta-analysis assimilating data from 8 studies presenting a marked elevation in COPD risk for RA patients compared to controls (RR = 1.82, 95% CI = 1.55–2.10). These findings are in synergy with our own results regarding the causal impact of RA on asthma or COPD.^[6]^

Though it was once widely accepted that RA was primarily associated with Th1 lymphocyte-induced inflammation, and asthma largely influenced by Th2 lymphocytes, the connection between diseases driven by these cell types was not clearly understood. This led to a prevailing notion that RA and asthma may co-occur in patients without sharing a direct biological relationship. Contrasting this older view, more recent studies have unveiled a prominent involvement of Th1 lymphocytes in the pathogenesis of asthma, particularly severe asthma.^[27]^ GWAS conducted by Li et al have discovered that cumulative genetic scores of SNPs within the genes IL12A, IL12RB1, STAT4, and IRF2, all part of the Th1 pathway, bear a negative association with forecasted FEV1 percentages and a positive 1 with the gravity of asthma symptoms.^[32]^ Adding to this, Raundhal et al undertook an examination of bronchoalveolar lavage cells from asthma patients ranging from mild to severe and discerned an intensified IFN-γ (Th1) immune response in those with severe asthma, alongside subdued Th2 and IL-17 responses.^[33]^ Th17 cells are recognized as fundamental players in the pathogenesis of RA.^[34]^ Evidence from asthma models in mice has demonstrated that IL-17A and IL-17F, cytokines produced by Th17 cells, are instrumental in prompting the proliferation and mobilization of mast cells, which are pivotally involved in orchestrating airway inflammation.^[35,36]^ These cytokines also exacerbate TH2-associated eosinophilia,^[37]^ and contribute to an enhanced state of airway hyperresponsiveness and Mucin5AC secretion.^[38,39]^ Observational studies have reported augmented levels of IL-17 in the respiratory tracts of individuals with asthma,^[40]^ proposing a conceivable link underlying the co-prevalence of RA and asthma. Moreover, anti-citrullinated protein antibodies (ACPAs), known to be intimately linked to the onset of RA,^[41,42]^ have also been correlated with pulmonary interstitial pathologies.^[43]^ Research by Alessandra et al uncovered an associative trend between heightened amounts of ACPAs and an incremented occurrence of asthma and COPD within female patients suffering from RA.^[44]^ These findings raise the possibility that RA could trigger the genesis of asthma and COPD via mechanisms driven by ACPA-related inflammation, a process that may even precede the clinical manifestation of joint symptoms.^[44]^ Presently, a prevailing hypothesis in the medical community posits that autoimmune and inflammatory reactions are central to the onset of COPD in patients with RA.^[31,45,46]^ This is chiefly characterized by a heightened production of autoantibodies that target a wide array of the body’s own proteins, alongside elevated levels of pro-inflammatory markers in the circulatory system of those afflicted with COPD.^[47–49]^ It is theorized that the inflammatory processes associated with RA may adversely impact the cells lining the alveoli in the lungs, which could lead to alveolar wall degradation and as a consequence, COPD.^[50]^

The results of this study have significant public health implications. First, we have revealed the genetic association between RA and both asthma and COPD. Second, it is recommended that routine pulmonary function tests be conducted in RA patients for early diagnosis of asthma or COPD. Third, timely treatment for RA patients is essential to prevent the occurrence of asthma and COPD.

Our MR study, which probed into the putative causative link between RA and obstructive pulmonary disorders such as asthma and COPD within The Japanese Population, encountered several constraints. Initially, the exclusive utilization of GWAS data from Japanese groups suggests that the inferences we reached may not be applicable to other ethnic demographics. Another concern pertains to the potential for bias––specifically, the winner’s curse and weak instrument bias^[26,51]^—stemming from the use of SNPs for both exposure and outcome derived from an identical sample set, possibly leading to an overestimation in the MR analytical results. Nonetheless, an existing empirical investigation has demonstrated that such biases are unlikely to exert a significant distortion on the findings.^[26]^ By incorporating a stringent GWAS significance cutoff in our sensitivity analysis, we ascertained that there was no discernible association between genetically forecasted RA and the likelihood of developing asthma or COPD. Further MR studies underscored by more granular and expansive GWAS data within East Asian and The Japanese Population are requisite for more conclusive results.

## 5. Conclusion

In summary, our study results suggest a potential causal relationship between genetically predicted RA and obstructive respiratory diseases, including asthma and COPD, within the Japanese Population. Screening for asthma and COPD among RA patients could have significant implications. Larger-scale studies are warranted to substantiate our findings and explore the specific mechanisms involved.

## Acknowledgments

We thank the Japan Biobank for providing publicly available genome-wide association study data in rheumatoid arthritis, asthma, and chronic obstructive pulmonary disease.

## Author contributions

**Conceptualization:** Lianguo Wu.

**Data curation:** Shaoning Shen.

**Formal analysis:** Hanbing Zeng.

**Investigation:** Hanbing Zeng.

**Resources:** Hao Wei.

**Software:** Hao Wei.

**Supervision:** Hao Wei.

**Writing – original draft:** Shaoning Shen.

**Writing – review & editing:** Lianguo Wu.
